# Effects of testosterone and vitamin D on fall risk in pre-frail hypogonadal men: a factorial design RCT

**DOI:** 10.1016/j.jnha.2024.100217

**Published:** 2024-03-28

**Authors:** Heike A. Bischoff-Ferrari, Melanie Kistler-Fischbacher, Stephanie Gaengler, Thomas Münzer, Bess Dawson-Hughes, Wei Lang, Robert Theiler, Andreas Egli, E. John Orav, Gregor Freystaetter

**Affiliations:** a Centre on Aging and Mobility, University of Zurich, Zurich, Switzerland; b Department of Aging Medicine and Aging Research, University of Zurich, Zurich, Switzerland; c IHU HealthAge, University Hospital Toulouse and University III Paul Sabatier, Toulouse, France; d Geriatrische Klinik St. Gallen, St. Gallen, Switzerland; e Jean Mayer USDA Human Nutrition Research Center on Aging, Tufts University, Boston, Massachusetts; f Department of Biostatistics, Harvard T.H. Chan School of Public Health, Boston, Massachusetts, USA

**Keywords:** Prevention, Vitamin D, Testosterone, Frailty, Falls

## Abstract

**Objective:**

To test whether transdermal testosterone at a dose of 75 mg per day and/or monthly 24’000 IU Vitamin D reduces the fall risk in pre-frail hypogonadal men aged 65 and older.

**Design:**

2 × 2 factorial design randomized controlled trial, follow up of 12 months.

**Methods:**

Hypogonadism was defined as total testosterone <11.3 nmol/L and pre-frailty as ≥1 Fried- frailty criteria and/or being at risk for falling at the time of screening. The primary outcomes were number of fallers and the rate of falls, assessed prospectively. Secondary outcomes were appendicular lean mass (ALM), sit-to-stand, gait speed, and the short physical performance test battery. Analyses were adjusted for age, BMI, fall history and the respective baseline measurement.

**Results:**

We aimed to recruit 168 men and stopped at 91 due to unexpected low recruitment rate (1266 men were pre-screened). Mean age was 72.2 years, serum total testosterone was 10.8 ± 3.0 nmol/l, and 20.9% had 25(OH)D levels below 20 ng/mL. Over 12 months, 37 participants had 72 falls. Neither the odds of falling nor the rate of falls were reduced by testosterone or by vitamin D. Testosterone improved ALM compared to no testosterone (0.21 kg/m^2^ [0.06, 0.37]), and improved gait speed (0.11 m/s, [0.03, 0.20]) compared to placebo.

**Conclusion:**

Transdermal testosterone did not reduce fall risk but improved ALM and gait speed in pre-frail older men. Monthly vitamin D supplementation had no benefit.

## 1 Introduction

Falls are among the leading causes of morbidity and mortality among older adults [1], posing a significant social and economic burden to individuals, health care systems and society [2]. One in three people age 65 years and older falls at least once each year and this proportion increases to 50% for those age 80 years and older [3]. In addition to established risk factors for falls such as age, muscle weakness, gait impairment, cognitive decline and vitamin D deficiency [4,5], sex hormone levels have been identified as a modifiable risk factor for falls in older men and women [6].

Several studies found that testosterone levels in men decrease with age [[8], [9], [10]]. Consistently, prevalence of hypogonadism (low testosterone levels) is estimated to be 20% for men over 60, 30% over 70 and 50% for men 80 years or older [8]. Moreover, the increase in hypogonadism has been found to be relevant for muscle function, with several studies suggesting an association with reduced muscle mass [11], reduced muscle strength [12], reduced mobility [13], and increased risk of falling [6,7,13].

On the other hand, higher physiological levels of total testosterone among community-dwelling men aged 65 years and older in the Boston Stop-It trial conferred a 78% decreased odds of falling when comparing the highest (≥14.2 nmol/l) to the lowest (≤9.4 nmol/l) physiological quartile range of serum total testosterone levels (OR = 0.22, 95% CI 0.07, 0.72) [6]. Notably, the decrease in the odds of falling among the men in the highest physiological quartile of total testosterone levels was most pronounced among men randomized to daily 700 IU vitamin D supplements (OR = 0.16, 95% CI, 0.03, 0.90) [6]. These data suggest a potential benefit of higher physiological total testosterone levels in combination with vitamin D supplementation.

While intervention trials on testosterone supplementation and fall risk as the primary outcome are missing, intervention trials in hypogonadal older men have consistently shown improved functional capacity (e.g., 6-minute walking distance) [14], muscle strength and power [15], as well as lean mass [15] with testosterone supplementation. In the Testosterone Trials, a set of three double-blind, 12-month randomized controlled trials with a total sample of 790 men aged 65 years and older with low serum testosterone (<9.5 nmol/l), a significantly greater proportion of men increased their 6-minute walking distance by ≥50 meters in the testosterone compared to placebo groups (OR = 1.77, *P* = 0.003) [14]. Furthermore, in a 3-year randomized controlled trial among 256 men (mean age: 67 years) with serum testosterone levels of 3.5–13.9 nmol/l, testosterone treatment compared to placebo improved chest muscle strength (mean difference 16.3 N [95% CI 5.5, 27.1]), chest and leg muscle peak power (22.5 W [7.5–37.5] and 83.8 W [35.4–132.2]) and increased lean mass (0.9 kg [0.5–1.4]) [15].

Mechanistically, the effects of testosterone on muscle are mediated by androgen receptor signaling, which regulates muscle structure, fiber type, intramyocellular metabolism and mRNA expression [16,17]. Additionally, testosterone promotes differentiation of pluripotent mesenchymal cells into myogenic lineage cells and inhibits differentiation into adipogenic lineage, supporting an increase in muscle mass and reduction in fat mass [16].

With regard to vitamin D, recent clinical trials and meta-analyses have supported the benefits of daily 800–1000 IU vitamin D on fall and fracture prevention among vulnerable older adults at risk of vitamin D deficiency [[18], [19], [20], [21]]. However, other studies have questioned the benefit of vitamin D for fall prevention among generally healthy adults age 50 years and older without vitamin D deficiency or osteoporosis [22,23], provided in daily supplements as tested in the VITAL [24] and DO-HEALTH [25] trials or in monthly applications in the ViDA [26] and the DO-HEALTH [27] trials. Mechanistically, a benefit of vitamin D on fall prevention has been suggested by the presence of the VDR in muscle tissue [28] and the observation of muscle weakness in vitamin D deficient older adults [29].

Therefore, the aim of the present trial was to test the individual and combined effects of daily testosterone supplementation and monthly vitamin D supplementation on fall risk among pre-frail, hypogonadal older men. We further examined the effect of testosterone and vitamin D individually and in combination on lean mass, muscle function and strength.

## 2 Methods

### 2.1 Study design

This was a 12-month, randomized, double-blind, placebo-controlled trial with a 2 × 2 factorial design, examining the effect of two interventions: (1) daily 75 mg of transdermal testosterone compared to daily placebo gel, and (2) monthly 24,000 IU vitamin D3 compared to monthly placebo. The 12-month study duration included 4 clinical visits (screening, baseline, 6 and 12 months) and 4 bi-monthly phone calls between clinical visits at 2, 4, 8 and 10 months of follow up. The maximum duration between screening and baseline visits was set to 28 days, due to the comprehensive screening assessments.

The trial took place at the Centre on Aging and Mobility, University of Zurich, Switzerland between October 2015 and May 2021. The protocol and statistical analysis plan was approved by the Ethics Commission of Zurich (KEK-ZH-Nr. 2014-0436) and prospectively registered on clinical trials.gov (NCT02419105).

### 2.2 Study participants

Pre-frail, hypogonadal older men were recruited through mailing lists, posters, flyers, public events, advertisements in newspapers and other media, and health care providers from the area of Zurich, Switzerland. Volunteers who expressed interest had to fulfill the following inclusion criteria: age 65 years and older; low serum total testosterone levels (<11.30 nmol/L); at higher risk for falling (fulfill at least 1 criteria of the Fried-based frailty criteria [30] and/or being at least at low risk for falling based on the adapted FROP-Com screening tool [31]). Additional inclusion and exclusion criteria are given in supplement Table S1.

**Fig. 1:**
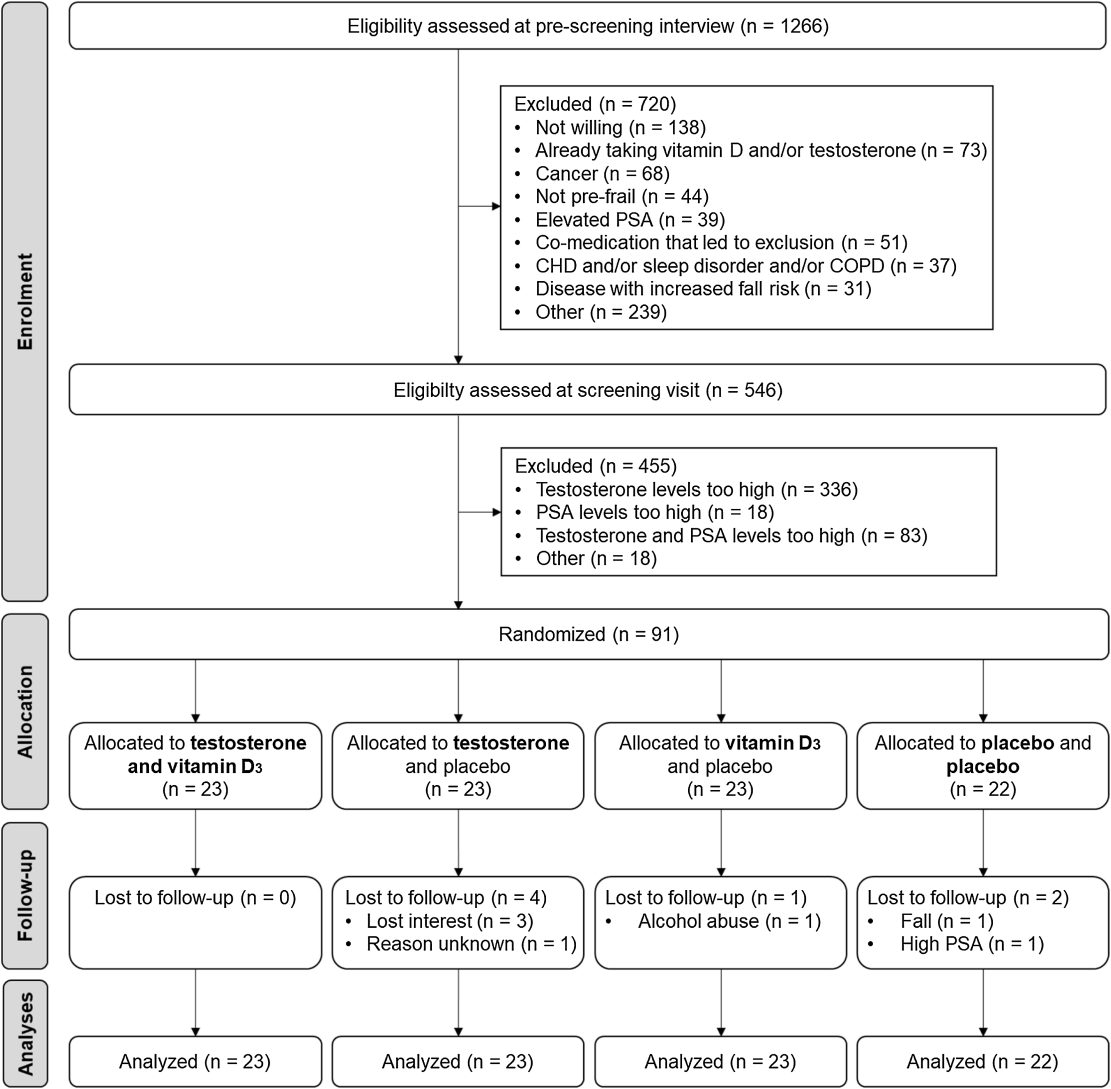
CONSORT diagram of participant flow.

The original inclusion criterion “low trauma fall in the last 12 months” was replaced by “at higher risk for falling”, 9 months after starting participant recruitment. This inclusion criterion was changed because the majority of prospective volunteers could not recall a fall event and therefore were excluded during the pre-screening interview. Fulfilling at least 1 criteria of the Fried-based frailty criteria and/or being at least at low risk for falling based on the adapted FROP-Com screening tool, still ensured enrolment of a pre-frail population at higher risk for falling, however, the criterion was multidimensional and not just focused on fall history alone. After changing this criterion the proportion of participants passing the pre-screening interview rose from 17% to 43% (Fig. 1).

### 2.3 Randomization and masking

Participants were block randomized (block size of 8 participants) to 1 of 4 treatment groups, stratified by age (<75 and ≥75 years) and fall history (at least one low trauma fall in the 12 months prior to enrolment: yes/no). The stratification variable “fall history” was included after changing the inclusion criteria, as described above. Randomization was computer-based and performed through the trial software (FDS randomization software, developed for DO-HEALTH [32,33]), which allowed treatment allocation and blinding.

The study medications were packed and labelled at the respective companies (see below) and individual kits were sent directly to the study center. Each kit looked identical, except for randomization codes. The information linking randomization codes to package content was stored by the trial software and inaccessible until study unblinding after trial completion. Participants and all investigators were blinded to group allocation.

### 2.4 Study interventions

The transdermal testosterone gel was transparent in appearance and contained 1.00% testosterone as the active substance plus isopropyl myristate, ethanol, carbomer, sodium hydroxide and water as excipients. The gel was applied daily in a dose of 6 full pump actions, which equated to a total dose of 75 mg of testosterone per day. The placebo transdermal gel had identical appearance, excipients, and application (6 pump actions daily) but contained 0.0% testosterone. Both, the testosterone and placebo gels were manufactured and provided by Besins Healthcare.

The vitamin D drinking solution was clear in appearance and contained 24,000 IU of vitamin D3 as the active substance plus polysorbate 20, purified water, glycerol, ethanol 96% and nitrogen as excipients. The 5-ml oral solution was ingested once per month. The placebo drinking solution had identical appearance, excipients, and application (5 ml drinking solution once monthly) but contained 0 IU vitamin D3. Both, the vitamin D and placebo drinking solutions were manufactured and provided by Dr. Wild & Co. AG.

### 2.5 Compliance

Compliance with the study interventions was assessed through accountability records, which were completed by the investigators. The quantities of study supplements dispensed to and returned by each participant were recorded at each clinical visit (baseline, 6 and 12 months). For the drinking solution, the number of full, partially full and empty bottles was counted. For the transdermal gel, compliance was calculated based on the weight difference between dispensed and returned gel tubes. Participants were considered compliant if they took at least 80% of the investigational product.

### 2.6 Measures

The two primary outcomes were the number of people who fell at least once and the rate of falls (any falls, low-trauma falls, injurious falls) during 12 months of follow-up. Falls were assessed at two clinical visits (6 and 12 months) and four bi-monthly phone calls (2, 4, 8, 10 months). Participants were asked about events happening in the previous two months of the visit/call. Falling was defined as unintentional coming to rest on the ground, floor or other lower level. Coming to rest against furniture or a wall was not considered a fall. Low-trauma falls were defined as falls from standing height, without the involvement of others. Injurious falls were defined as falls that led to any injury (e.g., skin wound, significant bruising, fracture). For the five participants for whom study medications were not available on the day of their baseline visit, incident falls were counted from the day they were randomized and received treatment and not from baseline.

Secondary outcomes included appendicular lean mass, lower extremity function and strength, and gait speed. Lower extremity function was assessed with the Short Physical Performance Battery (SPPB), which includes gait speed, five-times sit-to-stand and a balance test [34]. Each of the three components of the SPPB is scored 0–4 (4 indicating best performance) and are summed for a total score. Lower extremity strength and gait speed were measured using the five times sit-to-stand and 4-meter walk test, respectively, and were measured as time to complete the test in seconds. Both tests were part of the SPPB and measurements were taken at baseline, 6 and 12 months. Appendicular lean mass (ALM) was measured using Dual-energy X-ray Absorptiometry (Lunar DPX-NT, Lunar, General Electrics Healthcare, Munich Germany) at baseline and 12 months and adjusted for height [kg/m^2^]. The repeatability of the measurements were high with an intra-class-correlation-coefficient of 0.997, assessed in a subset of n = 27 participants.

Venous blood samples were taken in the morning, in a fasting state. Biomarkers used for screening purposes (e.g., total testosterone) were analyzed immediately after sample collection at the laboratory of the City Hospital Waid, Zurich. Total testosterone was analyzed using a Cobas 6000 analyzer module e601. Samples collected at baseline, 6 and 12 months were kept frozen (at −80°C) until trial completion and were then analyzed at the Institute of Clinical Chemistry (IKC) at the University Hospital Zurich. Total testosterone was analyzed using second generation electrochemiluminescence immunoassay (ECL) technology on a Cobas 8000 analyzer, module e801. Serum 25(OH)D was analyzed using LC-MS/MS on a 4500MD analyzer.

### 2.7 Adverse events

Adverse events (AEs) were recorded every two months, at the clinical visits and bi-monthly phone calls, and information on onset, duration, intensity, required treatment, outcome and action taken was collected. AEs were defined as any untoward medical occurrence in a patient which could be any unintended sign or symptom (including an abnormal laboratory finding), whether or not related to the study intervention. Serious AEs (SAEs) were defined as any untoward medical occurrence that: resulted in death; was life-threatening; required participant hospitalization or prolongation of hospitalization; resulted in persistent or significant disability/incapacity; or any important medical event which may jeopardize the subject or may require intervention to prevent one of the outcomes listed above.

The severity of each AEs was rated by a blinded physician as mild (hardly noticeable, negligible impairment of well-being), moderate (marked discomfort, but tolerable without immediate relief) or severe (overwhelming discomfort, calling for immediate relief). In addition, causality of the AE to the study treatment(s) was rated as unrelated, unlikely, possibly, probably or definitely.

Safety outcomes defined in the study protocol included incidence of cardiovascular events (myocardial infarction, stroke, revascularization procedures of CABG and PI, incident congestive heart disease, cardiovascular mortality) and events related to hemoglobin (>172 g/l), hematocrit (≥50 L/L), PSA (≥4.0 ng/ml, and/or increase of >1.4 ng/ml/year), eGFR (CKD-EPI equation, ≥−13.7 %) and liver enzyme levels. In addition, the International Prostate Symptom Score (IPSS) was assessed at every visit [35].

### 2.8 Sample size

The target sample size of the trial was N = 168, based on power calculations using the effect size from the Boston Stop-It trial, a 3-year RCT on the effect of vitamin D3 and calcium on the risk of falls [6]. In that study 38% of participants fell in the intervention group and 62% fell in the control group, resulting in a 24% difference. The observed rate of falls for the same period was 0.9 (SD 2.2) per person-year in the intervention group and 1.9 (SD 1.7) per person-year in the control group (difference: RD = 1.0; ratio RR = 2.1) (unpublished data). Assuming a dropout rate of 17%, based on our experience, the trial had 82% power to detect a difference in the number of participants sustaining at least one fall and 90% power to detect a difference in rate of falls.

The trial was stopped prematurely after enrolment of 91 participants. The main reason for early termination was the low recruitment rate (7%), due to the unexpectedly large number of participants with testosterone levels ≥11.30 nmol/l (336 [74%] out of 455 screened participants). Further reasons for the low recruitment rate were lack of willingness to perform and comply with the study-related procedures, elevated PSA, already taking vitamin D and/or testosterone. Based on this, continuation of the trial was not deemed feasible and the trial was terminated prematurely, in accordance with the Cantonal Ethics Commission of Zurich and the Swiss surveillance authority for medicines and medical device Swissmedic.

At the time when early termination of the trial was discussed, a revised power calculation was performed for the primary outcome and for the secondary outcome gait speed, based on a sample size of 90 participants. No group differences was expected for the primary outcome with the reduced sample size, as the power was 76%. For the secondary outcome, these revised calculations yielded 85% power to detect a difference of 0.2 m/s in gait speed, assuming an interaction of treatment effects (n = 21 per group) and a 7% drop out rate. The calculations were based on existing data from the ongoing trial (e.g., adherence to clinical visits, standard deviations for gait speed from actual study participants [0.24 m/s]) and on data from previous studies [14,36].

### 2.9 Statistical analysis

Baseline characteristics of the study population were described by treatment groups. Normally distributed continuous variables are presented as mean and standard deviation (SD) and non-normally distributed variables as median and interquartile range (IQR). Categorical variables are presented as frequencies and percentages. The treatment effects on the odds of falling at least once were analyzed using logistic regression, adjusting for age (continuous age and an indicator of age >75 years), body mass index (BMI), fall history and person-time. The rate of falls was analyzed using negative binomial regression with the same covariates. As there were no significant interactions between treatments, main effects of vitamin D and testosterone were presented, along with the comparison between the group with the combined intervention versus the group with no intervention. In our sensitivity analyses the number of falls per participant was capped at 4.

For secondary outcomes, separate mixed effects models were fit for SPPB, fivetimes sit-to-stand and gait speed to examine treatment, time, and treatment by time interaction effects on the changes from baseline to 6 and 12 months accounting for repeated measurements within participant using a unstructured covariance structure. The model was adjusted for age (continuous age and an indicator of age >75 years), fall history, BMI and baseline value of the outcomes. For ALM, which was measured at baseline and 12 months, analysis of variance model was fit to examine treatment effects on change in ALM from baseline to 12 months, adjusted for age (continuous age and an indicator of age >75 years), fall history, BMI and baseline ALM. Except for gait speed, there were no significant interactions between treatments and therefore main effects of vitamin D and testosterone were examined in the models. For gait speed, due to the significant interaction effect of vitamin D-by-testosterone, all four arms were compared to each other.

Pre-specified secondary analyses for the primary outcomes were by achieved quartiles of 25(OH)D and total testosterone.

All analyses were conducted according to the intention-to-treat principle and included all participants who were randomized and received the study intervention package at baseline. For the continuous outcomes missing data were imputed using multiple imputation with n = 10 rounds. Analyses were conducted using SAS version 9.4 (SAS Institute Inc., Cary, NC, USA) with a significance level of 0.025.

## 3 Results

### 3.1 Study participants

Of 1126 volunteers interested in the trial, 553 met the pre-screening inclusion criteria, and of those 91 men met the blood level targets to be enrolled in the trial (Fig. 1; recruitment rate was 7%). Mean age was 72.2 ± 5.9 years and 49 (53.8%) participants reported a history of falling in the 12 months prior to enrolment. The mean baseline 25(OH)D concentration was 26.8 ± 7.6 ng/mL and 20.9% had a 25(OH)D level of less than 20 ng/mL. The mean total testosterone concentration at baseline was 10.8 ± 3.0 nmol/L. Participants in the vitamin D group had higher BMI (P = 0.023) and lower albumin (P = 0.007) compared to the no-vitamin D group, but there were no other differences between treatment groups (Table 1).

**Table 1:** Participant demographic characteristics.

	Overall (n = 91)	No-vitamin D (n = 45)	Vitamin D (n = 46)	No testosterone (n = 45)	Testosterone (n = 46)
Prior fall, n (%)	49 (53.8)	23 (51.1)	26 (56.5)	24 (53.3)	25 (54.3)
Age, mean (SD)	72.2 (5.9)	72.4 (5.9)	72.0 (5.9)	71.8 (5.3)	72.5 (6.4)
Living situation, n (%)					
Alone	15 (16.5)	5 (11.1)	10 (21.7)	4 (8.9)	11 (23.9)
Other	1 (1.1)	0 (0.0)	1 (2.2)	1 (2.2)	0 (0.0)
Partner	6 (6.6)	4 (8.9)	2 (4.3)	3 (6.7)	3 (6.5)
Spouse	69 (75.8)	36 (80.0)	33 (71.7)	37 (82.2)	32 (69.6)
BMI [kg/m^2^], mean (SD)	26.8 (3.0)	26.1 (3.0)	27.5 (2.9)	26.5 (2.9)	27.1 (3.2)
Years of education [years], median (IQR)	15.0 (13.0, 17.0)	15.0 (12.0, 17.0)	15.0 (13.0, 17.0)	15.0 (13.0, 17.0)	15.0 (13.0, 17.0)
Former smoker, n (%)	55 (60.4)	26 (57.8)	29 (63.0)	30 (66.7)	25 (54.3)
Number of comorbidities, median (IQR)	1.0 (0.0, 2.0)	1.0 (0.0, 1.0)	1.0 (0.25, 2.0)	1.0 (0.0, 2.0)	1.0 (0.25, 2.0)
Sangha Comorbidity score, median (IQR)	2.0 (0.0, 4.0)	2.0 (0.0, 3.0)	2.0 (0.25, 4.0)	2.0 (0.0, 3.0)	2.0 (0.25, 4.0)
Albumin [g/L], mean (SD)	45.3 (2.0)	45.9 (1.9)	44.7 (2.1)	44.9 (1.9)	45.7 (2.1)
25(OH)D [ng/mL], mean (SD)	26.8 (7.6)	27.4 (7.7)	26.1 (7.6)	26.4 (7.9)	27.1 (7.4)
Vitamin D deficiency (25(OH)D <20 ng/mL), n (%)	19 (20.9)	7 (15.6)	12 (26.1)	11 (24.4)	8 (17.4)
SHBG [nmol/L], mean (SD)	41.0 (16.1)	40.6 (13.6)	41.3 (18.3)	42.7 (16.2)	39.3 (15.9)
PSA [ng/mL], median (IQR)	1.22 (0.74, 2.08)	1.20 (0.87, 2.06)	1.29 (0.62, 2.06)	1.42 (0.75, 2.51)	1.19 (0.73, 1.68)
Hemoglobin [g/L], mean (SD)	148.40 (10.06)	149.11 (10.21)	147.70 (9.98)	147.93 (10.04)	148.85 (10.17)
Hematocrit [L/L], mean (SD)	0.44 (0.03)	0.44 (0.03)	0.44 (0.03)	0.44 (0.03)	0.44 (0.03)
Total testosterone [nmol/L], mean (SD)^a^	10.8 (3.0)	11.1 (3.0)	10.4 (2.9)	11.0 (2.3)	10.5 (3.5)
Free testosterone [nmol/L], mean (SD)	0.19 (0.06)	0.19 (0.06)	0.18 (0.06)	0.19 (0.05)	0.19 (0.06)

Abbreviations: BMI - body mass index, PSA - Prostate-specific Antigen, SHBG - Sex Hormone Binding Globulin, 25(OH)D - 25-hydroxyvitamin D.

a Testosterone concentrations reported here were measured at baseline. The measurement at screening visit was used to determine whether inclusion criteria were met.

Seven (8%) participants dropped out from the trial during the 12-month intervention period. One participant dropped out between the baseline and 6 month visits, and the other six participants dropped out between 6 and 12 month visits. The reasons for dropping out were loss of interest (n = 3), alcohol abuse (n = 1), a fall that led to hospitalization (n = 1), high PSA (n = 1) and withdrawal of consent without giving a reason (n = 1). Compliance with the study interventions across the 12 months of follow-up was 78.0 ± 30.6% for the drinking solution, with no differences for placebo versus vitamin D (78.3 ± 28.6% versus 77.7 ± 32.8%, P = 0.924). For the transdermal gel, compliance was 57.3 ± 32.5% overall, with higher adherence for those receiving placebo compared to testosterone (64.3 ± 34.2% versus 50.4 ± 29.4%, P = 0.040), see Supplemental Table S2. The achieved concentrations of testosterone and vitamin D3 at baseline and over time are presented in Supplemental Table S3.

### 3.2 Falls

Odds of falling: A total of 72 falls occurred in 37 (41%) participants and 33 injurious falls in 24 (26%) participants. There were no differences in the odds of being a faller for testosterone versus no testosterone (OR = 1.10, 95% CI 0.44, 2.73), vitamin D versus no vitamin D (OR = 2.56, 95% CI 0.97, 6.73) or vitamin D + testosterone versus placebo (OR 2.81, 95% CI 0.72, 10.98; Table 2). Also, no benefit by any treatment was seen for the odds of being an injurious faller (Table 2).

**Table 2:** Effects of treatments on the odds of being a faller and injurious faller.

	Vitamin D (vs. no vitamin D)	Testosterone (vs. no testosterone)	Testosterone + vitamin D (vs. placebo)
Being a faller			
OR (95% CI)	2.243 (0.891, 5.650) P = 0.086	1.100 (0.456, 2.654) P = 0.833	2.467 (0.672, 9.052) P = 0.173
Being an injurious faller			
OR (95% CI)	1.678 (0.608, 4.632) P = 0.318	0.727 (0.268, 1.973) P = 0.531	1.220 (0.292, 5.103) P = 0.785

Adjusted for age, BMI, fall history, person-time.

Rate of falling: There were no significant differences in fall rates for any of the treatments (Table 3), including subgroups of low trauma and injurious falls.

**Table 3:** Effects of treatments on fall risk.

	Vitamin D		Testosterone		Combination	
	Vitamin D	No vitamin D	Testosterone	No testosterone	Testosterone + vitamin D	Placebo
Total falls						
Incidence rate (95% CI), person-years	0.88 (0.52–1.48)	0.59 (0.33–1.03)	0.59 (0.332–1.05)	0.87 (0.53–1.43)	0.76 (0.36–1.60)	0.74 (0.37–1.48)
Incidence rate ratio (IRR) (95% CI)	1.45 (0.75–2.82)		0.68 (0.34–1.33)		1.01 (0.39- 2.64	
P value	0.252		0.255		0.982	
Low trauma falls						
Incidence rate (95% CI), person-years	0.79 (0.47–1.34)	0.51 (0.29−0.92)	0.51 (0.28−0.92)	0.82 (0.50–1.35)	0.68 (0.32–1.437)	0.68 (0.34–1.35)
IRR (95% CI)	1.54 (0.77–3.09)		0.64 (0.32–1.27)		0.99 (0.38–2.595)	
P value	0.222		0.203		0.981	
Injurious falls						
Incidence rate (95% CI), person-years	0.46 (0.28−0.77)	0.27 (0.14−0.52)	0.30 (0.16−0.56)	0.42 (0.25−0.71)	0.37 (0.17−0.81)	0.30 (0.13−0.69)
IRR (95% CI)	1.69 (0.79–3.62)		0.72 (0.34–1.51)		n/a^a^	
P value	0.176		0.382		n/a^a^	

Adjustment for age, BMI and fall history, person-time.

a Model did not converge.

For the two sensitivity analyses, results remained robust if number of falls per participant were capped at 4.

Results from pre-defined analyses by achieved quartile of total testosterone at month 12 revealed no differences in fall rate by achieved testosterone levels (Supplemental Table S4). For achieved 25(OH)D levels at month 12, participants in the highest achieved quartile of 25(OH)D (37.5–46.6 ng/mL) had a significantly increased fall rate compared to the lowest quartile (IRR = 2.47, 95% CI 1.0, 5.9, P = 0.044; lowest quartile range: 7.9–26.0 ng/mL; Supplemental Table S6).

### 3.3 Functional performance

**Fig. 2:**
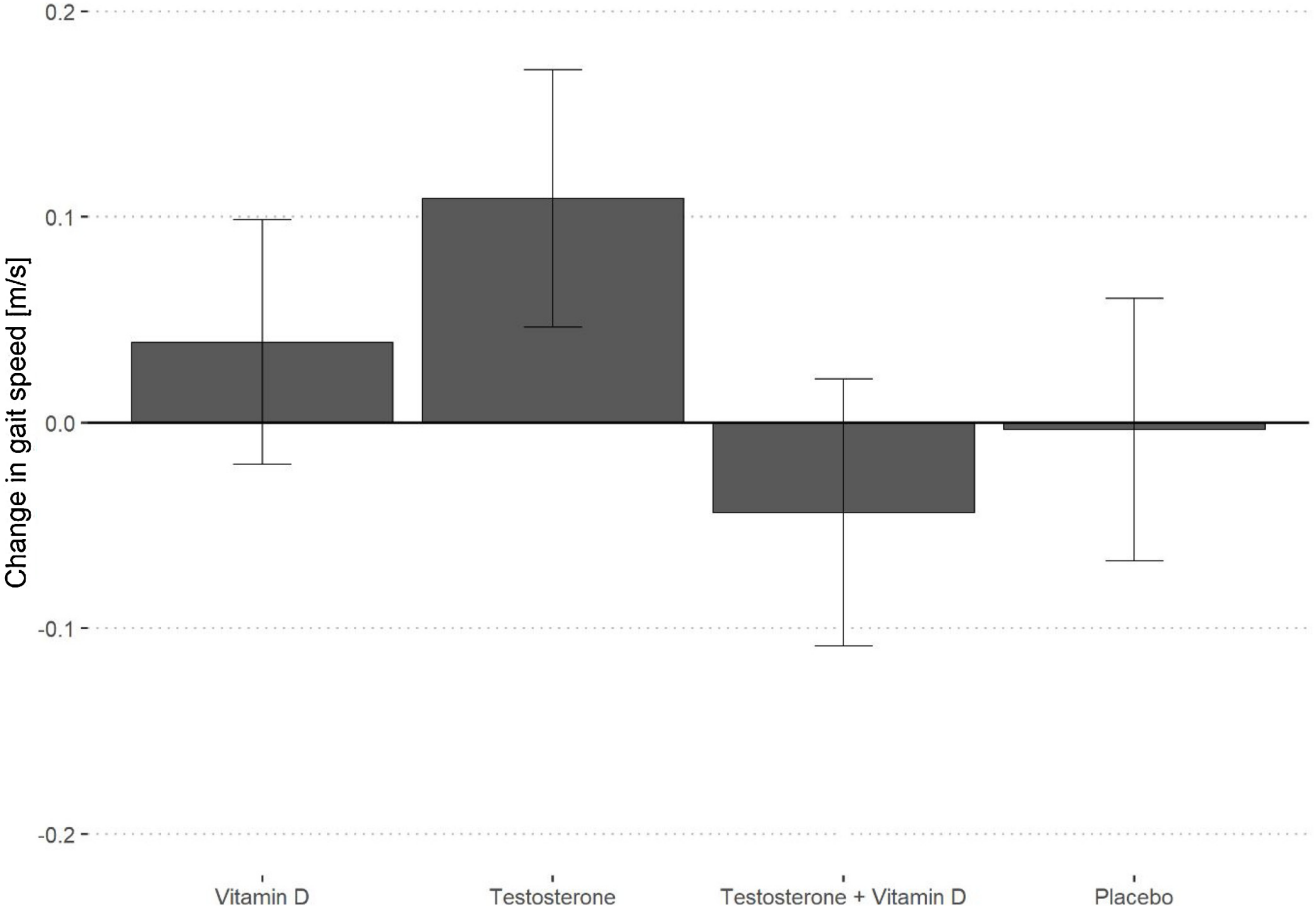
Adjusted least square means (LSMs) for 12-month changes in gait speed by treatment group. Legend Fig. 2: LSM obtained from the mixed effects models adjusting for age, BMI and baseline gait speed. Error bars represent 95% confidence intervals. P values for vitamin D = 0.303, testosterone = 0.010; combination = 0.352.

For the SPPB or 5-times sit-to-stand test (Table 4), there were no between-group differences in changes from baseline. For gait speed, the testosterone + placebo group experienced a significant improvement compared to the placebo group (difference in adjusted least square means [LSM] = 0.11 m/s, 95% CI 0.028, 0.197, P = 0.010; Fig. 2). The sensitivity analysis with multiple imputation of the missing observations (7.7%) did not show significant differences (Supplemental Table S4).

**Table 4:** Effects of treatments on SPPB and lower extremity strength from linear mixed effects models.

	Vitamin D	No vitamin D	P value	Testosterone	No testosterone	P value
SPPB [points]						
Baseline, mean (SD)	11.4 (1.2)	11.7 (0.6)		11.4 (1.2)	11.7 (0.6)	
Change BL - 6 M, LSM^a^ (95% CI)	−0.15 (−0.34; 0.05)	−0.03 (0.22; 0.17)	0.341	−0.17 (−0.43; 0.09)	−0.05 (−0.31; 0.21)	0.863
Change BL - 12 M, LSM^a^ (95% CI)	−0.17 (−0.43; 0.08)	−0.05 (−0.32; 0.22)	0.500	−0.08 (−0.27; 0.12)	−0.10 (−0.29; 0.1)	0.493
Sit-to-stand test [seconds]						
Baseline, mean (SD)	9.6 (2.4)	9.4 (2.2)		9.6 (2.3)	9.4 (2.4)	
Change BL - 6 M, LSM^a^ (95% CI)	0.16 (−0.50; 0.81)	−0.41 (−1.07; 0.26)	0.186	0.07 (−0.59; 0.72)	−0.32 (−0.96; 0.33)	0.358
Change BL - 12 M, LSM^a^ (95% CI)	0.17 (−0.56; 0.90)	−0.33 (−1.09;0.42)	0.302	0.19 (−0.55; 0.93)	−0.36 (−1.09; 0.38)	0.255

Abbreviations: LSM – Least square mean, BL - Baseline, M - Months, SPPB - Short Physical Performance Battery.

a LSM obtained from the mixed effects models adjusting for age, BMI, fall history and baseline level of the outcome.

### 3.4 Appendicular lean mass

**Fig. 3:**
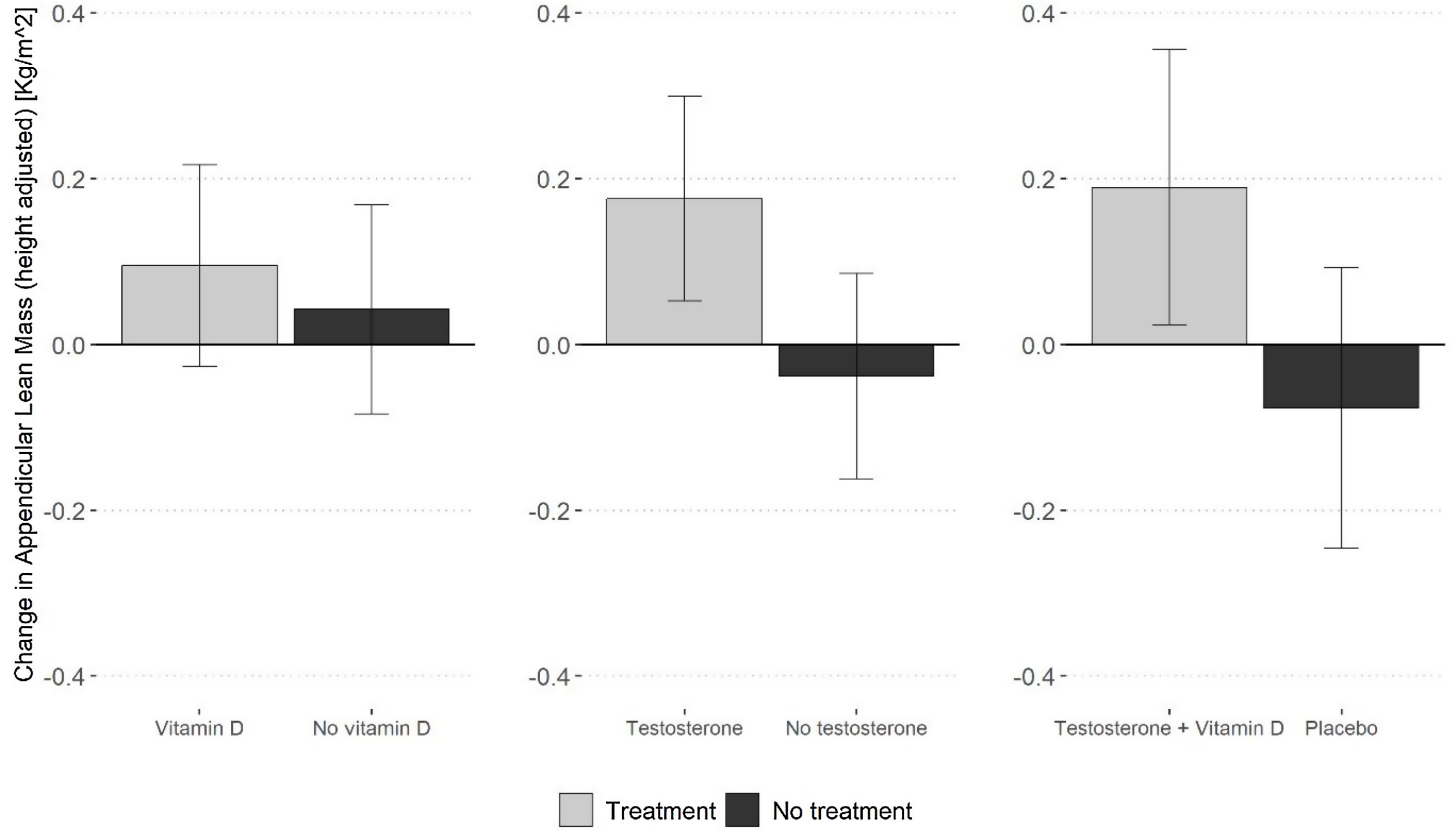
Adjusted least square means (LSMs) for 12-month changes in ALM by treatment group. Legend Fig. 3: N = 82, LSM were obtained from the analysis of variance model adjusting for age, BMI and baseline ALM level. P values for vitamin D = 0.497; testosterone = 0.007; combination = 0.036.

Testosterone compared to no testosterone (LSM = 0.212 kg/m^2^, 95% CI 0.061, 0.364) and combined testosterone + vitamin D compared to placebo (LSM = 0.262 kg/m^2^, 95% CI 0.046, 0.478) increased ALM (Fig. 3). Vitamin D had no effect on ALM (LSM = 0.050 kg/m^2^, 95% CI −0.102, 0.202).

### 3.5 Safety

There were 263 adverse events (AEs) and 15 serious adverse events (SAEs) during the trial (Supplemental Table S7). The number of AEs and SAEs did not differ between treatment groups. Three SAEs were classified as possibly related to testosterone treatment (SADR), but only 1 (hypertensive crisis) of them happened in a participant treated with testosterone. With respect to the predefined safety outcomes, out of 22 participants with elevated hematocrit and/or hemoglobin, 17 received testosterone versus 5 did not receive testosterone. Regarding the prostate gland, 6 participants in the testosterone group versus 3 in the non-testosterone group showed an increase of PSA of more than 1.4 ng/mL/year and/or the PSA value increased to more than 4 ng/mL. In contrast, only 15 participants receiving testosterone versus 20 participants not receiving testosterone showed an increase in IPSS, which corresponds to stronger symptoms of benign prostatic hyperplasia. The eGFR (CKD-EPI equation) decreased by at least 13.7 % in 4 participants in the testosterone group versus 7 in the non-testosterone group. There was one participant not receiving testosterone with elevated liver enzymes and no AE due to elevated serum calcium or cardiovascular events (Supplemental Table S7).

## 4 Discussion

In this randomized factorial design trial among 91 pre-frail hypogondal older men, both daily testosterone supplementation and monthly vitamin D or their combination did not reduce the odds of falling or the rate of falls. However, we cannot exclude that our trial was under-powered for the primary outcome on falls as the trial aimed to reach a recruitment of 168 participants and was terminated early due to challenges in recruitment. For the secondary outcomes of function and muscle mass, the trial supports a significant improvement of gait speed and appendicular lean mass with testosterone supplementation over the 12-month follow-up, but no benefit with vitamin D.

Only one previous trial investigated the effect of testosterone supplementation on incident falls among 380 men aged 65 years and older with baseline serum testosterone levels <9.5 nmol/L [37,38]; however, the men in that trial were not selected for level of frailty. That trial tested a daily application of a transdermal testosterone gel for 12 months. The initial dose was 50 mg/day with a target to reach and maintain serum levels of 500−800 ng/dL (17.3–27.7 nmol/L) and the dose was adjusted based on 3-montly measurements of serum testosterone levels [38]. Consistent with our findings, transdermal testosterone had no effect on the fall rate [37]. In a pre-defined sensitivity analysis of our trial, fall risk also did not differ by achieved testosterone blood levels at 12 months.

Our findings that monthly vitamin D supplementation had no effect on fall reduction or function in largely vitamin D replete community-dwelling older men is in line with the recent literature [22,23,39]. Our findings are also consistent with several trials suggesting that intermittent bolus dosing of vitamin D is either ineffective in healthy older adults or detrimental in vulnerable older adults [40]. Notably, in this trial, we did observe a significant increase in fall risk among men who achieved the highest (37.5–46.6 ng/mL) quartile of 25(OH)D levels at 12 months compared to men who achieved the lowest quartile level (7.9–26.0 ng/mL).

Regarding our finding that testosterone supplementation improved appendicular muscle mass, we also align with previous RCTs and meta-analyses among generally healthy [[41], [42], [43]], pre-frail [44,45] and frail [45] older men supporting a benefit of testosterone supplementation on muscle mass. Additionally, we found that testosterone supplementation improved gait speed in pre-frail hypogonadal men, and changes in gait speed were within the proposed range of clinically important differences of 0.1–0.2 m/s [46]. Thus, our findings support benefits of testosterone supplementation on gait speed, consistent with the Testosterone Trials which found improvements in the 6-minute walking test [14]. The documented benefit of testosterone supplementation on gait speed did however not translate to benefits in lower extremity function on SPPB. This may have been due to ceiling effects; the participants had almost maximum baseline SPPB levels.

In our trial, compliance with the transdermal gel was 57%, which is similar to compliance reported in another 12-month randomized controlled trial in frail hypogonadal men (54%) [44], but lower than in several other trials of transdermal gel application (>85%) [37,45,47]. In contrast, however, data from cohort studies indicate much lower compliance with testosterone treatment (e.g., 15–17% [48,49]).

Regarding pre-defined safety outcomes, adverse events and serious adverse events were balanced across testosterone and no testosterone groups. However, there was a higher number of participants with elevated hematocrit and/or hemoglobin levels in the groups receiving testosterone compared to those not receiving testosterone treatment (17 vs. 5). Similarly, more participants in the testosterone group had elevated and/or increased PSA levels (6 vs. 3), whereas the IPSS did not increase in the testosterone groups. We did not observe an increased risk of serious adverse events or cardiovascular events for testosterone versus no testosterone groups, which aligns with the literature [50]. It is known that testosterone treatment increases hematocrit and hemoglobin [50] by stimulating erythropoiesis and thus inducing erythrocytosis [51]. In the present trial, testosterone treatment led to an increase in hematocrit and/or hemoglobin in 17/46 (37%) of participants. For comparison, in the Testosterone Trials, one year of testosterone treatment was associated with a hemoglobin rise above 17.5 g/dL in 7 out of 394 participants (2%) while the number of participants with increased hematocrit were not reported [14]. This proportion is considerably lower compared to our trial, despite very similar thresholds (17.5 and 17.2 g/dL). In the Testosterone Trials, elevated PSA levels were more common in the testosterone group, an observation that was confirmed in our trial [14].

Our trial had several strengths. First, to the best of our knowledge, this is the first trial that examined the effect of testosterone supplementation on falls in pre-frail older hypogonadal men with or without additional vitamin D supplementation. Also, the trial investigated both functional measures and muscle mass, and a series of pre-defined safety endpoints for both treatments.

Our trial also has limitations. First, recruitment of study participants with serum total testosterone levels below the set threshold of ≤ 11.3 nmol/L posed a significant challenge and the desired sample size of 168 participants could not be achieved, despite manifold recruitment strategies. Notably, both the chosen threshold of hypogonadism [14,45,47,52,53] in our trial and the low recruitment rate of 7% (e.g., Borst et al.,: threshold of <10.4 nmol/L, recruitment rate of 5% [53]; Amory et al.,: threshold of <12.1 nmol/L, recruitment rate of 10% [52]) aligns with current literature. Second, only 21% of participants were vitamin D deficient at baseline, which reduced the trial’s chance to detect fall reduction by vitamin D supplementation. Third, analyses were not adjusted for multiple comparison and chance findings can thus not be excluded. Finally, the study included pre-frail, hypogonadal men and findings may thus not be applicable to general healthy, or frailer individuals.

In summary, transdermal testosterone improved gait speed and increased appendicular lean mass; however, it did not reduce falls. Our results may be limited by the small sample size and a larger clinical trial is therefore needed to clarify the role of transdermal testosterone in fall prevention among pre-frail hypogonadal men. Vitamin D given monthly did not improve fall risk or function in largely vitamin D replete pre-frail hypogonadal men.

## Funding

The trial was funded by the Swiss National Science Foundation (SNSF, grant number 32003B_135192). Further Besins Healthcare and Dr. Wild & Co. provided the study medication (testosterone gel/placebo, and vitamin D3/placebo, respectively) and both provided independent funding.

## Conflict of interest

Heike A. Bischoff-Ferrari: Prof. Bischoff-Ferrari is the PI of the T&D trial. Industry partners Besins and Wild provided the study medication (testosterone gel/placebo, and vitamin D3/placebo, respectively) and both provided independent funding.

Melanie Kistler-Fischbacher: has nothing to declare.

Stephanie Gaengler: has nothing to declare.

Thomas Münzer: has nothing to declare.

Bess Dawson-Hughes: has nothing to declare.

Wei Lang: has nothing to declare.

Robert Theiler: has nothing to declare.

Andreas Egli: has nothing to declare.

E. John Orav: has nothing to declare.

Gregor Freystaetter: has nothing to declare.

The authors declare that they have no known competing financial interests or personal relationships that could have appeared to influence the work reported in this paper.
