# Supplementary material for: Effects of testosterone and vitamin D on fall risk in pre-frail hypogonadal men: a factorial design RCT
Source: J Nutr Health Aging. 2024 Mar 28;28(5):100217. doi: 10.1016/j.jnha.2024.100217 (PMC12877218; doi:10.1016/j.jnha.2024.100217)
Supplement: Supplementary file 1 [file mmc1.docx]

# SUPPLEMENTAL FILE

**Effects of testosterone and vitamin D on fall risk in pre-frail hypogonadal men: a factorial design RCT**

Heike A. Bischoff-Ferrari^1,2,3^, Melanie Kistler-Fischbacher^1,2^, Stephanie Gaengler^1,2^, Thomas Münzer^4^, Bess Dawson-Hughes^5^, Wei Lang^1,2^, Robert Theiler^2^, Andreas Egli^1,2^, E. John Orav^6^, Gregor Freystaetter^1,2^

## Supplemental Table 1: Additional inclusion and exclusion criteria.

| **Additional inclusion criteria** |
| --- |
| - able to come to the study center - BMI > 18.0 and < 35.0 kg/m^2^ - understand German (reading and writing) sufficient to understand and complete questionnaires and tests - willingness to limit calcium supplementation to 500 mg/day and vitamin D supplementation to 800 IU/day - willing to forgo active vitamin D metabolites and testosterone products for the duration of the trial - Mini Mental State Examination Score of > 24 - willing and able to perform and comply with study-related procedures. |
| **Additional exclusion criteria** |
| - contraindications to study treatments - treatment with vitamin K-antagonists, insulin, adrenocorticotropic hormone or corticosteroids - elevated PSA levels (≥ 4 ng/ml) and/or palpable signs of prostate or breast cancer - low hemoglobin (≤ 100 g/l) - high hematocrit (≥ 0.50 L/L) - liver function values more than 3 times the upper limit of normal - elevated serum calcium (≥ 2.60 mmol/l) - low creatinine clearance (≤ 30 ml/min) - severe visual or hearing impairment - history of cancer <5 years prior to signing informed consent, except for adequately treated basal cell or squamous cell skin cancer - myocardial infarction in the last 3 months, unstable angina pectoris and/or exertional dyspnea ≥ NYHA III - other severe medical conditions (e.g., epilepsy, chronic pulmonary disease, uncontrolled hypertension end-stage diseases; alcohol abuse; severe gait impairment) - participation in another interventional trial in the 6 months prior to screening - enrolment of family members, employees or other dependent person - Individuals who took more than 800 IU of vitamin D on any day in the 6 months prior to enrolment were also excluded. A prospective participant could be enrolled if the mean intake in the previous 6 months and since the last dose was ≤ 800 IU/day |
| **Additional temporary exclusion criteria** |
| - Individuals who took more than 800 IU of vitamin D on any day in the 6 months prior to enrolment were also excluded. A prospective participant could be enrolled if the mean intake in the previous 6 months and since the last dose was ≤ 800 IU/day |

## Supplemental Table 2: Compliance with the study medications

|  | Drinking solution | | | | Transdermal gel | | | |
| --- | --- | --- | --- | --- | --- | --- | --- | --- |
|  | Overall  (N = 91) | No vitamin D (n = 45) | Vitamin D  (n = 46) | P-value | Overall  (N = 91) | No testosterone  (n = 45) | testosterone  (n = 46) | P-value |
| **Mean compliance** | | | | |  | | | |
| BL - 6M, mean (SD) | 85.90 (33.40) | 84.44 (34.71) | 87.32 (32.38) | 0.684 | 67.33 (38.18) | 69.60 (37.37) | 65.11 (39.24) | 0.578 |
| 6M - 12M, mean (SD) | 70.15 (42.57) | 72.22 (40.51) | 68.12 (44.84) | 0.648 | 47.22 (44.22) | 59.01 (44.61) | 35.69 (41.10) | 0.011 |
| BL - 12M, mean (SD) | 78.02 (30.60) | 78.33 (28.56) | 77.72 (32.79) | 0.924 | 57.28 (32.45) | 64.31 (34.18) | 50.40 (29.42) | 0.040 |
| **Number of participants with ≥ 80% compliance** | | | | |  | | | |
| BL - 6M, mean (SD) | 71 (78.0) | 35 (77.8) | 36 (78.3) | 1.000 | 40 (44.0) | 21 (46.7) | 19 (41.3) | 0.761 |
| 6M - 12M, mean (SD) | 55 (60.4) | 27 (60.0) | 28 (60.9) | 1.000 | 21 (23.1) | 14 (31.1) | 7 (15.2) | 0.121 |
| BL - 12M, mean (SD) | 56 (61.5) | 28 (62.2) | 28 (60.9) | 1.000 | 21 (23.1) | 15 (33.3) | 6 (13.0) | 0.041 |

## Supplemental Table 3: Testosterone and vitamin D_3_ concentrations at baseline and over time by treatment groups.

| **Main treatment group** |  | **No treatment** |  |  | **Treatment** |  |  |
| --- | --- | --- | --- | --- | --- | --- | --- |
|  |  | Baseline | Month 6 | Month 12 | Baseline | Month 6 | Month 12 |
| Testosterone | 25-hydroxyvitamin D3[µg/L] | 26.43 (7.94) | 29.32 (9.29) | 32.02 (9.48) | 27.07 (7.40) | 29.42 (8.14) | 30.26 (7.17) |
|  | Total testosterone [nnom/L] | 0.19 (0.05) | 0.19 (0.06) | 0.18 (0.05) | 0.19 (0.06) | 0.35 (0.21) | 0.30 (0.24) |
|  | Free testosterone[nnmol/L] | 11.01 (2.29) | 11.43 (2.67) | 10.87 (2.73) | 10.52 (3.49) | 19.17 (12.70) | 15.61 (12.35) |
| Vitamin D_3_ | 25-hydroxyvitamin D3[µg/L] | 26.43 (7.94) | 29.32 (9.29) | 32.02 (9.48) | 27.07 (7.40) | 29.42 (8.14) | 30.26 (7.17) |
|  | Total testosterone [nnom/L] | 0.19 (0.05) | 0.19 (0.06) | 0.18 (0.05) | 0.19 (0.06) | 0.35 (0.21) | 0.30 (0.24) |
|  | Free testosterone[nnmol/L] | 11.01 (2.29) | 11.43 (2.67) | 10.87 (2.73) | 10.52 (3.49) | 19.17 (12.70) | 15.61 (12.35) |

*Supplemental Table 4: Sensitivity analysis of the secondary outcomes SPPB, five-times repeated sit- to-stand and gait speed using multiple imputation with (n=10)*

|  | **Difference (95%CI)** |  | **Difference (95%CI)** |  | **Difference (95%CI)** |  |
| --- | --- | --- | --- | --- | --- | --- |
| **Outcome** | **Testosterone** | **p-value** | **Vitamin D** | **P-value** | **Testosterone+ Vitamin D** | **P-value** |
| Overall change in SPPB (points) | 0.02 (-0.22; 0.26) | 0.862 | -0.12 (-0.36; 0.12) | 0.339 |  |  |
| Overall change in Sit-to-stand (seconds) | 0.36 (-0.44; 1.17) | 0.377 | 0.42 (-0.29; 1.36) | 0.206 |  |  |
| Overall change in gait speed* (seconds/meter) | 0.06 (-0.11; 0.23) | 0.480 | 0.04 (-0.12; 0.21) | 0.621 | 0.09 (0.04; 1060000000) | 0.138 |
| *the 4 Level variable was used dues to treatment interaction | | | | | | |

## Supplemental Table 5: Stratified negative binomial regression results by achieved quartiles of total serum testosterone at month 12

| **Outcome** | **Measure** | **Quartile 1** | **Quartile 2** | **Quartile 3** | **Quartile 4** | **Linear trend** |
| --- | --- | --- | --- | --- | --- | --- |
| **Falls** | Testosterone [nmol/L] | [1.91; 8.78] | [8.92; 11.20] | [11.40; 13.80] | [14.10; 57.90] |  |
|  | Incidence rate (95%CI) | 0.81  (0.34– 1.91) | 0.79  (0.39 – 1.59) | 1.00  (0.46 – 2.19) | 0.67  (0.32 - 1.42) | p=0.836 |
|  | IRR (95%CI) | Ref. | 0.97  (0.33 - 2.82) | 1.24  (0.41 – 3.70) | 0.83  (0.28 – 2.45) |  |
|  | P-value |  | 0.953 | 0.706 | 0.741 |  |
| **Injurious falls** | Incidence rate (95%CI) | 0.25  (0.10-0.61) | 0.38  (0.19-0.79) | 0.58  (0.29-1.16) | 0.34  (0.16-0.75) | p=0.557 |
|  | IRR (95%CI) | Ref. | 1.56  (0.49-4.92) | 2.34  (0.73-7.53) | 1.39  (0.42-4.68) |  |
|  | P-value |  | 0.453 | 0.154 | 0.591 |  |
| *Models included adjustments for age, prior fall, BMI* | | | | | | |

## Supplemental Table 6: Stratified negative binomial regression results by achieved quartiles of total serum 25(OH)D at month 12

| **Outcome** | **Measure** | **Quartile 1** | **Quartile 2** | **Quartile 3** | **Quartile 4** | **Linear trend** |
| --- | --- | --- | --- | --- | --- | --- |
| **Falls** | 25(OH)D [ng/mL] | [7.90; 26.00] | [26.10; 31.00] | [31.20; 37.30] | [37.50; 46.60] |  |
|  | Incidence rate (95%CI) | 0.64  (0.32-1.28) | 0.68  (0.34-1.35) | 0.49  (0.22-1.08) | 1.58  (0.89-2.81) | p=0.043 |
|  | IRR (95%CI) | Ref. | 1.051  (0.39-2.84) | 0.762  (0.262-2.22) | 2.47  (1.02-5.94) |  |
|  | P-value |  | 0.922 | 0.619 | 0.044 |  |
| **Injurious falls** | Incidence rate (95%CI) | 0.28  (0.12-0.65) | 0.36  (0.17-0.77) | 0.31  (0.14-0.71) | 0.56  (0.30-1.05) | p=0.226 |
|  | IRR (95%CI) | Ref. | 1.27  (0.40-4.02) | 1.11  (0.34-3.63) | 2.00  (0.71-5.62) |  |
|  | P-value |  | 0.680 | 0.870 | 0.188 |  |
| *Models included adjustments for age, prior fall, BMI* | | | | | | |

## Supplemental Table 7: Pre-defined safety outcomes for testosterone versus no testosterone treatment

|  | **Testosterone**  (n = 46) | **No Testosterone**  (n = 45) |
| --- | --- | --- |
| Total adverse events | 130 | 133 |
| Total serious adverse events | 5 | 10 |
| Total serious adverse drug reactions | 1^1^ | 0 |
| Elevated hematocrit (≥ 0.50 L/L) | 8 | 3 |
| Elevated hemoglobin (>172 g/L) | 0 | 0 |
| Elevated hematocrit (≥ 0.50 L/L) and elevated hemoglobin (>172 g/L) | 9 | 2 |
| Elevated PSA (≥ 4 ng/ml) | 1 | 3 |
| Increased PSA (>1.4 ng/ml/year) | 3 | 0 |
| Elevated PSA (≥ 4 ng/ml) and increased PSA (>1.4 ng/ml/year) | 2 | 0 |
| Increased in IPSS score^2^ | 15 | 20 |
| Decreased eGFR (CKD-EPI equation, ≥ -13.7 %) | 4 | 7 |
| Elevated liver enzymes (more than 3 times the upper limit of normal) ^3^ | 0 | 1 |
| Elevated calcium (≥2.60 mmol/L) | 0 | 0 |
| Cardiovascular events | 0 | 0 |
| ^1^ Hypertensive crisis (known hypertensive disease)  ^2^ Higher score means more symptoms  ^3^ 3 times the upper limit of normal for ALAT = 150 U/L; for ASAT = 150 U/L; for γ-GT = 213 U/L; for alkPhos = 390 U/L | | |
